# Selective anti-tumor activity of the novel fluoropyrimidine polymer F10 towards G48a orthotopic GBM tumors

**DOI:** 10.1007/s11060-013-1321-1

**Published:** 2013-12-18

**Authors:** William H. Gmeiner, Carla Lema-Tome, Denise Gibo, Jamie Jennings-Gee, Carol Milligan, Waldemar Debinski

**Affiliations:** 1 Department of Cancer Biology, Wake Forest School of Medicine, Winston-Salem, NC 27157 USA; 2 Department of Neurosurgery, Wake Forest School of Medicine, Winston-Salem, NC 27157 USA; 3 Department of Neurobiology and Anatomy, Wake Forest School of Medicine, Winston-Salem, NC 27157 USA; 4 Brain Tumor Center of Excellence, Wake Forest School of Medicine, Winston-Salem, NC 27157 USA

**Keywords:** Glioblastoma, Thymidylate synthase, Topoisomerase 1, Intra-cerebral drug administration, EphA2

## Abstract

F10 is a novel anti-tumor agent with minimal systemic toxicity in vivo and which displays strong cytotoxicity towards glioblastoma (GBM) cells in vitro. Here we investigate the cytotoxicity of F10 towards GBM cells and evaluate the anti-tumor activity of locally-administered F10 towards an orthotopic xenograft model of GBM. The effects of F10 on thymidylate synthase (TS) inhibition and Topoisomerase 1 (Top1) cleavage complex formation were evaluated using TS activity assays and in vivo complex of enzyme bioassays. Cytotoxicity of F10 towards normal brain was evaluated using cortices from embryonic (day 18) mice. F10 displays minimal penetrance of the blood–brain barrier and was delivered by intra-cerebral (i.c.) administration and prospective anti-tumor response towards luciferase-expressing G48a human GBM tumors in nude mice was evaluated using IVIS imaging. Histological examination of tumor and normal brain tissue was used to assess the selectivity of anti-tumor activity. F10 is cytotoxic towards G48a, SNB-19, and U-251 MG GBM cells through dual targeting of TS and Top1. F10 is not toxic to murine primary neuronal cultures. F10 is well-tolerated upon i.c. administration and induces significant regression of G48a tumors that is dose-dependent. Histological analysis from F10-treated mice revealed tumors were essentially completely eradicated in F10-treated mice while vehicle-treated mice displayed substantial infiltration into normal tissue. F10 displays strong efficacy for GBM treatment with minimal toxicity upon i.c. administration establishing F10 as a promising drug-candidate for treating GBM in human patients.

**Electronic supplementary material:**

The online version of this article (doi:10.1007/s11060-013-1321-1) contains supplementary material, which is available to authorized users.

## Introduction

**Fig. 1:**
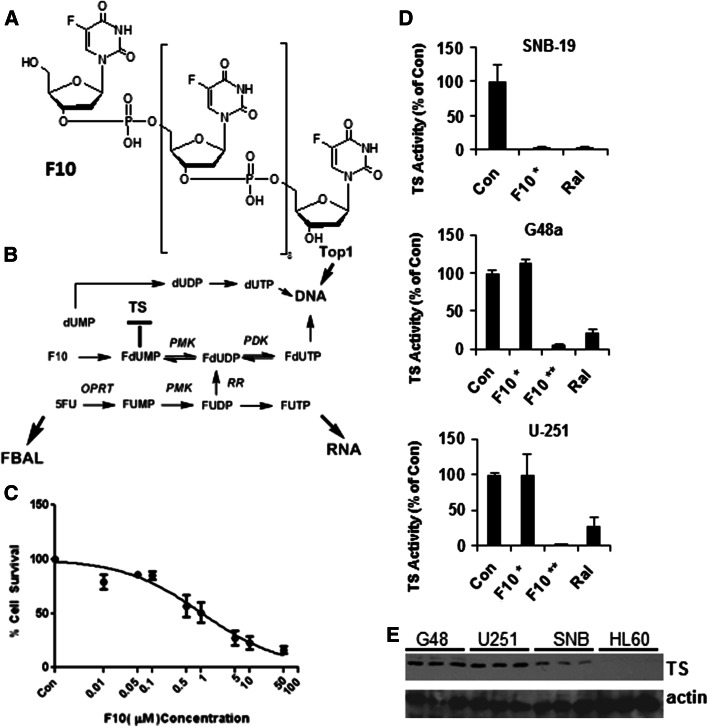
F10 is cytotoxic to GBM cells through induction of thymineless death. **a** Chemical structure of F10; **b** Metabolic conversion of F10 produces FdUMP and initiates Topoisomerase 1-mediated DNA double strand breaks; **c** Cytotoxicity of F10 to G48 cells; **d** TS inhibitory activity of F10 towards SNB-19 (*top*), G48a (*middle*), and U-251 (*bottom*) cells at 10^−6^ (*asterisk*) and 10^−5^ M (*double asterisk*) and for raltitrexed at 10^−6^ M. **e** Western blots (for three independent samples) evaluating TS expression in G48a, U-251, and SNB-19 cells relative to HL60 cells that are sensitive to F10 at nM concentrations. Overall sensitivity to F10 inversely correlates with TS expression

Glioblastoma (GBM) is the most common malignant brain tumor and one of the deadliest human malignancies [1, 2]. Optimal therapy results in survival times of ~15 months for newly diagnosed cancer and 5–7 months for recurrent disease [3]. New therapeutic modalities are urgently needed. We recently demonstrated that the novel fluoropyrimidine (FP) anti-tumor agent F10 (Fig. 1a) displayed strong anti-leukemic activity towards genetically-engineered syngeneic murine models [4] of acute myeloid [5] and acute lymphoid leukemia [6] that replicate the poor response of human patients to chemotherapy. F10 is a polymer of 5-fluoro-2′-deoxyuridine-5′-*O*-monophosphate (FdUMP), the thymidylate synthase (TS) inhibitory metabolite of 5-fluorouracil (5-FU). Anti-leukemic activity with F10 is achieved with markedly reduced systemic toxicities relative to the current treatment [7]. As F10 also displays strong cytotoxicity towards the central nervous system (CNS) malignancies included in the NCI 60 cell line panel [8], we sought to determine to what extent F10 would be effective for treating human GBM in an orthotopic setting and to what extent F10 cytotoxicity would differentiate between normal brain and malignant tissue.

The present studies address to what extent the high sensitivity of GBM cell lines to F10 in vitro can be harnessed to achieve strong anti-tumor activity towards a realistic animal model of GBM in vivo. We utilized an orthotopic xenograft model of GBM in which *luc*-transfected G48a GBM cells were injected directly into the brains of immunocompromised mice. The G48a cell line was established by our (WD) laboratory from cells isolated from a patient with multi-foci GBM [9, 10]. Intra-cerebral (i.c.) injection of G48a cells results in formation of a highly invasive and highly proliferative malignancy that replicates the characteristic features of the human disease. F10 does not readily penetrate the blood–brain barrier (BBB); however we report that i.c. administration of F10 results in dramatic regression of G48a tumors. The results demonstrate that the high sensitivity of F10 towards CNS malignancies in the NCI 60 cell line screen may be successfully translated into an efficacious in vivo treatment.

## Materials and methods

### Ethical statement

All animal experiments were performed in accordance with protocols approved by the Wake Forest School of Medicine Animal care and Use Committee in accordance with National Institutes of Health guidelines.

### Cell culture and ICE bioassay

G48a, U-251 and SNB-19 cells were grown in RPMI 1640 supplemented with 10 % FBS and glucose (2 g/L). In vivo complex of enzyme (ICE) bioassays were completed using methods previously described [11]. Cell samples were counted and equalized for cell number. Primary antibody (mouse anti-human DNA Top I, BD Pharmingen) was added at 1:500 dilution. Secondary antibody (Cell Signaling) was used at 1:1,000. ECL Lightning-Plus (PerkinElmer) was used for detection of the Topoisomerase 1 cleavage complex (Top1CC).

### TS catalytic activity assays

GBM cells were plated at a density of 1.5 × 10^6^ cells in 100 mm^2^ plates. Cells were grown overnight in RPMI 1640 medium with 10 % FBS. Cells were treated with 5-FU, F10, or raltitrexed at the indicated concentrations and incubated for 0–48 h, harvested, and lysed by freeze-fracturing. Following centrifugation of cell lysates, supernatants were assayed for protein content and TS catalytic activity as previously described [12]. Thymidine (Thy)-rescue was accomplished by adding Thy at 80 μM as the 20 μM dose previously described [13] was not effective.

### Caspase activity and viability assays

Caspase 3/7, 8, and 9 activity and cell viability assays were performed using Promega Caspase-Glo and Cell-Titer Glo Luminescent Assay reagents. Briefly, 2 × 10^5^ cells were plated in 24 well plates in triplicate and drug treatments started 20–24 h after plating. Cells were re-suspended before 50–100 μL aliquots were taken at the indicated times and mixed with an equal volume of assay kit reagent in a 96-well white plate. The plates were then incubated at RT for 30–60 min and visualized using a Tecan Genios luminescence plate reader. Apoptosis data was normalized for cell number using viability data [14].

### Intracranial GBM model

G48a-luc cells were suspended into Hanks Balanced Salt Solution to a density of 10^5^ cells/μL. Nude (nu/nu) mice (7-week old) were obtained from NCI. Mice were anesthetized using ketamine/xylazine and placed on a small-animal head-holding frame. A scalp incision was made to determine the drill-hole location, 1 mm right and 1 mm anterior to lambda. A 27-gauge needle attached to a 10 μL sterile Hamilton syringe (Hamilton, Reno, NV) was stereotactically inserted 3 mm below the dural surface and cells were injected into the deep white matter of the posterior thalamus. Cells were injected in a total of 5 μL over a 5 min period. The syringe was removed 2 min after injection was complete. After removal of the syringe, the hole was covered with cranio-plastic liquid. Animals were sutured, allowed to recover on a heating pad, and returned to their cages. Animal weight and behavior was monitored every 3 days.

### Animal groups and treatments

Starting on day 21 after tumor cell implantation, mice were treated by i.c. infusion (0.5 μL/h) of F10 at three concentrations (80, 120 and 160 mg/kg) for a duration of 7 days. Drug was prepared in phosphate buffered saline. Osmotic pumps (Alzet model 1007D, Alzet, Cupertino, CA) were primed in sterile saline overnight at 37 °C according to manufacturer’s specification. The pumps were then coupled to brain infusion kits (Alzet model 3). Animals were anesthetized using ketamine/xylazine and placed on a small-animal head holding frame, and the location of initial injection was retraced. The cannula was then inserted to a depth of 3 mm and secured with cranio-plastic adhesive and the pump was placed subcutaneously between the shoulder blades. Animals were sutured, allowed to recover and evaluated for any motor deficits resulting from the surgical procedure. Each treatment group had five animals.

### IVIS imaging

Tumor growth was monitored by evaluating bioluminescence [15] using the IVIS Lumina II imaging system (Xenogen Corporation, Alameda, CA). Animals received an intraperitoneal (i.p.) injection of d-luciferin (150 mg/kg, stock solution 15 mg/mL in sterile PBS, Goldbio, St. Louis, MO). After 10 min, animals were anesthetized with isofluorane until non-responsive, and then placed in the imaging chamber. Three bioluminescent imaging [15] acquisitions were collected at different exposure times (10, 60, 300 s), at the end of the study measurements at a exposure time of 10 s were chosen for the analysis, as the other exposures resulted in overexposure of some images that would have affected the accuracy of the results. Mice were allowed to recover and returned to their cages. Data were analyzed based on total photon flux emission (photons) in the region of interest over the intracranial space using Living Image software (Xenogen Corp.). The average of the change in total photon per treatment group at the end of the study was compared using a t-test comparing each treatment group against vehicle (Prism, GraphPad Software, La Jolla, CA).

### Tissue processing and immunohistochemistry

Animals were deeply anesthetized with an i.p injection of ketamine/xylazine and fixed by transcardial perfusion through the heart with PBS followed by 4 % paraformaldehyde in PBS (PFA). Brains were removed and fixed overnight in 4 % PFA. Brains were then cryoprotected in 30 % sucrose-PBS at 4 °C for 2–3 days. Brains were embedded in tissue-freezing medium (Triangle Biomedical Sciences, Durham, NC) and sections were cut to a thickness of 10 μm, thawed onto SuperFrost Plus slides (Fisher, Pittsburgh, PA) and kept at −20 °C until further processing as described in supplementary methods.

### Cortical neuronal cultures

Cortical neuronal cultures were prepared using previously described protocols [16] and described in Supplementary Methods. Results are expressed as percent control (mean ± SD) where control represents cultures without the addition of 5-FU or F10 (*n* = 4 individual experiments with at least two wells per condition). Statistical significance was determined with a one-way ANOVA followed by the Bonferroni post-hoc test.

## Results

### F10 is cytotoxic towards GBM cells

We evaluated the cytotoxic mechanism of F10 towards G48a, SNB-19, and U-251 cells to gain insight into the processes by which this novel FP polymer may be effective for GBM treatment. Although not exceptionally sensitive to F10, the G48a cell line (IC_50_ ~ 1 × 10^−6^ M; Fig. 1c) was selected for further study in vivo because G48a cells form a highly invasive and rapidly growing tumor upon injection into the brains of immunocompromised mice [9]. This orthotopic GBM model replicates several of the challenging features associated with treatment of the human disease in that it is highly infiltrative and rapidly growing.

### F10 targets TS and Top1 in GBM cells

We evaluated TS activity and the ability of exogenous thymidine (Thy) to rescue the cytotoxic effects of F10 to determine to what extent F10-induced thymineless death towards GBM cells (Fig. 1). F10 inhibited TS completely in G48a, U-251, and SNB-19 GBM cells (Fig. 1d), although higher F10 concentrations were required for G48a and U-251 cells than in previous studies [5, 12]. The anti-folate Raltitrexed [17] also effectively inhibited TS in these GBM cells (Fig. 1d). Western blots revealed a strong inverse correlation between TS expression and F10 sensitivity (Fig. 1e). Thymidine (Thy)-rescue experiments [13] demonstrated exogenous Thy reversed F10 cytotoxicity during the first 18 h of treatment (supplementary Fig. 1a) but was not effective at later time points (supplementary Fig. 1b, c). Top1 cleavage complexes (Top1CC) were also detected in GBM cells following F10 treatment (supplementary Fig. 1d, e) and exogenous Thy was able to reverse Top1CC formation only if provided prior to DNA replication. The results are consistent with F10 cytotoxicity arising from the dual targeting of Top1 and TS in GBM cells.

### F10 displays minimal toxicity towards primary cortical neurons

**Fig. 2:**
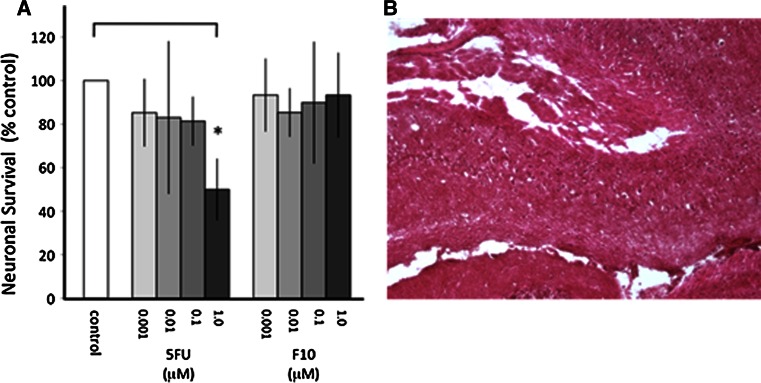
F10 is not toxic to primary neuronal cultures and does not damage normal brain upon i.c. administration. **a** Viability of primary neuronal cells grown in tissue culture following treatment with F10 or 5-FU at the indicated doses. 5-FU, but not F10, significantly decreased neuronal survival at 1 μM relative to control (*p* < 0.05). ANOVA followed by Newman–Keuls multiple comparison test: control versus 1 μM 5-FU; *p* ≤ 0.05; 1 μm 5-FU versus 1 μM F10; *p* ≤ 0.05. **b** H&E stained section from the brain of mice treated with F10 at 120 mg/kg

Strong differential cytotoxicity towards malignant relative to non-malignant cells is critical for effective cancer treatment. To gain insight into the sensitivity of normal brain tissue to F10, we evaluated the cytotoxicity of F10 towards primary cortical neuronal cultures from mice. We also evaluated 5-FU that has been shown to be toxic to normal neuronal cells [18]. The results are shown in Fig. 2. F10 treatment at concentrations as high as 1 μM resulted in no significant reduction in viability for primary neurons while 5-FU treatment (1 μM) resulted in ~50 % decreased viability. The 1 μM dose for F10 exceeded the GI_50_ value for all CNS malignancies included in the NCI 60 cell line panel. Thus, F10 displays a substantial therapeutic window with preferential cytotoxicity towards malignant cells.

### F10 is well-tolerated upon i.c. administration and efficacious for GBM

F10 does not penetrate the BBB in healthy mice (data not shown), thus intra-cranial (i.c.) administration of F10 results in high local concentrations that may be therapeutically beneficial. Dose-finding studies in nude mice demonstrate that F10 administered i.c. using an Alzet osmotic mini-pump at doses up to 200 mg/kg administered over 7 days are well-tolerated and do not cause damage to normal brain (Fig. 2b). The total dose administered in these studies is considerably less than used in recent leukemia studies evaluating systemic treatment (200 mg/kg/dose × 4 doses over 7 days) [5], consistent with F10 being retained within the BBB upon i.c. administration. At a dose of 200 mg/kg over 7 days mice displayed mild light-sensitivity and were somewhat lethargic. No serious morbidities were observed with F10 treatment.

**Fig. 3:**
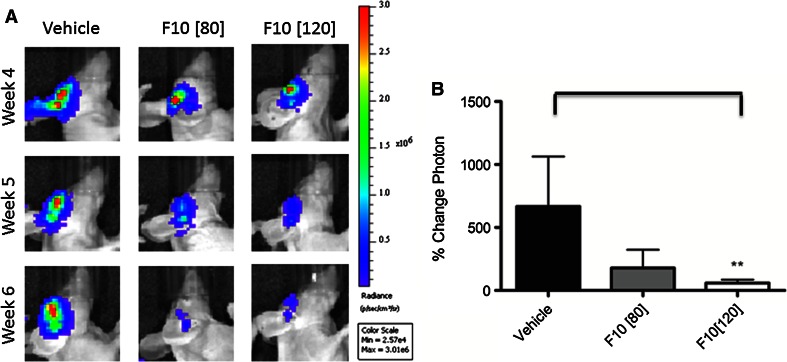
F10 treatment results in significant and dose-responsive regression of G48a orthotopic xenografts. **a** Luciferase signal from F10- and vehicle-treated nude mice at 80 and 120 mg kg doses. Treatment with F10 results in marked decrease in luciferase-signal for treated mice. **b** Mean tumor luminescence calculated from the luciferase images. F10 treatment results in regression of G48a xenografts that is highly significant (*p* < 0.01) relative to vehicle for the 120 mg/kg treatment

F10 efficacy was evaluated using G48a orthotopic xenografts in nude mice. F10 was administered at 80, 120, and 160 mg/kg over 7 days and anti-tumor activity was evaluated by IVIS imaging (Fig. 3a). The results for the 80 and 120 mg/kg treatment are summarized in Fig. 3b. Based on the luminescence signal, tumors grew rapidly in mice receiving vehicle-only with mean tumor volumes increasing ~600% over the course of the study. In contrast, i.c. administered F10 resulted in significant tumor regression (Fig. 3b). Mean tumor luminescence (measured by %-change in photon emission) for mice treated with 120 mg/kg F10 were significantly decreased relative to control (*p* < 0.01). All mice receiving F10 treatment displayed tumor regression. Interestingly, mice treated with 160 mg/kg F10 did not display luminescent signal decreased to the extent observed for the other treatment groups due to drug-related effects on luminescence imaging. Inspection of tissues, however, indicated a strong dose-dependent anti-tumor response for all F10 doses.

### F10 is selectively cytotoxic towards GBM cells in vivo

**Fig. 4:**
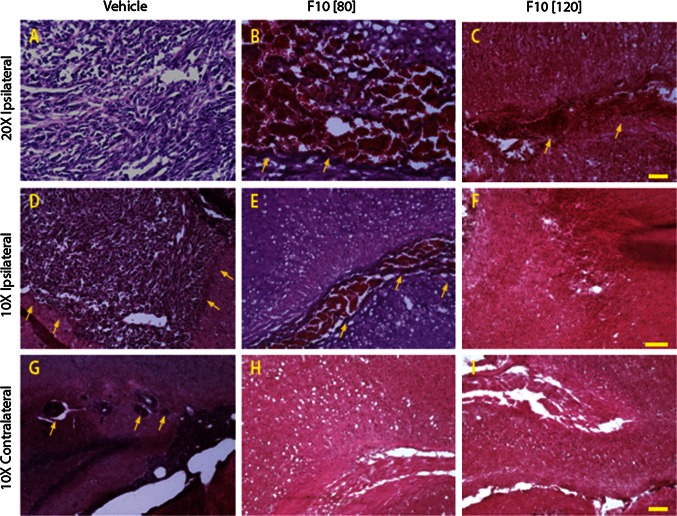
F10 treatment results in selective eradication of G48a orthotopic brain tumors in nude mice. H&E staining from brain sections obtained from nude mice bearing G48a xenografts following treatment with vehicle (**a**, **d**, **g**) or F10 at 80 (**b**, **e**, **h**) or 120 mg/kg (**c**, **f**, **i**). F10, or vehicle, was administered i.c. over 7 days using an osmotic pump. Brain tissue from mice treated with vehicle-only revealed extensive infiltration of GBM cells into non-malignant tissue and a significant tumor mass (*arrows* point to tumor borders). Treatment with F10 at either 80 or 120 mg/kg resulted in extensive necrosis selectively for malignant cells with no apparent damage for non-malignant cells in the contralateral side at the level of the hippocampus. Invasive island of cells were observed in contralateral hippocampus of vehicle-treated animals (*arrows* in **g**) (*Scale bar* 100 μm in **c** and **i**, 50 μm in **f**)

Histological examination of brain tissue revealed that F10 treatment caused selective death of GBM cells with no apparent damage to normal brain tissue (Fig. 4a, d). G48a cells produced highly infiltrative and rapidly growing tumors in the brains of immunocompromised mice with tumors localized to the hemisphere where cells were injected. H&E staining of brain sections from vehicle- and F10-treated mice demonstrated that F10 treatment resulted in extensive areas of necrosis selectively within tumor tissue and only in the tumor-bearing side of the brain (Fig. 4a–f). The extent of F10-induced necrosis was dose-dependent with the 120 mg/kg dose (Fig. 4c, f) inducing greater tumor cell necrosis than the 80 mg/kg dose (Fig. 4b, e). There was no observable necrosis in the contralateral side of brains from F10-treated mice (Fig. 4g–i).

**Fig. 5:**
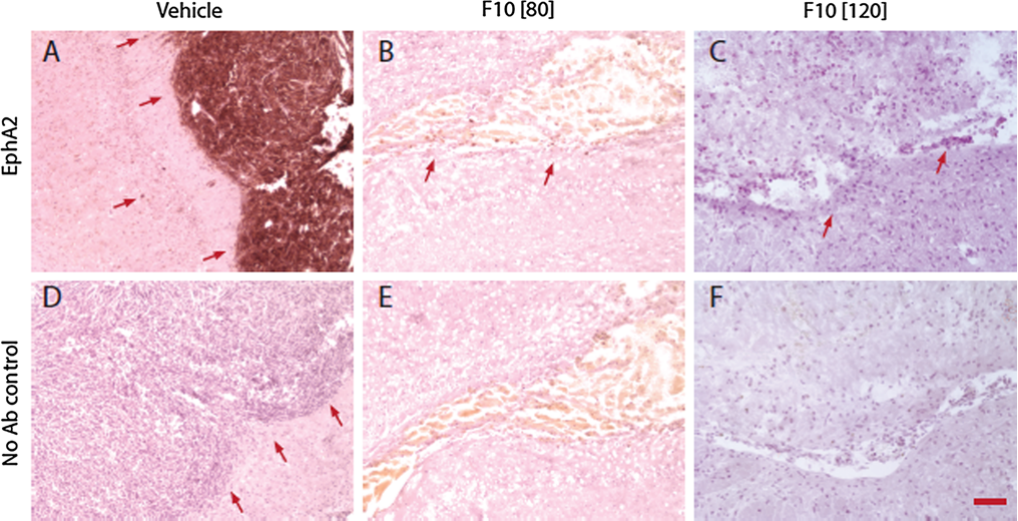
F10 treatment results in selective eradication of EphA2-stained G48a cells. EphA2 staining from brain sections obtained from nude mice bearing G48a xenografts following treatment with vehicle only (**a**, **d**) or F10 at 80 (**b**, **e**) or 120 mg/kg (**c**, **f**). Strong EphA2 staining is observed for tumor tissue in vehicle-only treated mice with no EphA2 staining in adjacent non-malignant tissue except in isolated invasive cells (*arrows* indicate tumor borders and point to invasive cells away from tumor core). EphA2 staining is greatly diminished in region of the residual tumor (indicated by *arrows*) from mice treated with F10 at 80 mg/kg and is absent in mice treated with F10 at 120 mg/kg (*Scale bar* 50 μm)

To further validate selective death of malignant cells in vivo we performed EphA2-staining of brain sections (Fig. 5). Previous studies have established EphA2 as a brain tumor specific antigen [19] that is useful for tumor-targeting as well as tumor imaging. EphA2 staining of tissues from vehicle-only treated mice revealed extensive regions of tumor mass in the tumor-bearing side of the brain (Fig. 5a) while the contralateral side did not display any EphA2-positive cells. In contrast, sections from the tumor-bearing side of mice treated with F10 at 120 mg/kg showed no EphA2 staining, even in the necrotic tissue that was the remnants of the malignant mass while only trace levels of EphA2 positive staining were detected in the ventricle of mice treated with F10 at 80 mg/kg (Fig. 5b, c). As with vehicle-only treated mice, no EphA2 staining was detected in the contralateral sides of brains from F10-treated mice.

## Discussion

Analysis of data from the NCI 60 cell line screen indicated cells derived from human CNS malignancies were particularly sensitive to F10 treatment with nanomolar potency towards several cell lines (e.g. SF268) and a remarkably large differential sensitivity for F10 relative to 5-FU (~10,000-fold for several GBM cell lines [8]). In the present studies, we evaluated the anti-tumor activity of F10 towards a G48a orthotopic xenograft model of GBM. GBM is particularly challenging for treatment, in part, because GBM cells are highly invasive and rapidly proliferating. We selected G48a cells for in vivo studies because these cells form orthotopic tumors in nude mice and the resulting tumors display the aggressive, infiltrating characteristics of the human disease. Our results demonstrate that F10 administered i.c. is not only highly effective at reducing the growth rate of G48a xenografts in vivo, but that F10 actually induces significant tumor reduction (Fig. 3). Tumor reduction was achieved in a dose-dependent manner with histological analysis indicating extensive necrosis (Fig. 4) and essentially complete tumor eradication based on elimination of EphA2 staining (Fig. 5). Importantly, this dramatic anti-tumor effect was achieved with no apparent damage to normal brain tissue (Fig. 4) demonstrating the potential for F10 to be administered via convection enhanced delivery (CED) in patients. CED [20, 21] is a minimally invasive technique of delivering drugs directly to brain tumors. A Phase 3 trial of IL-13 toxic conjugate showed equivalency to standard of care demonstrating the feasibility of CED for effective treatment [22].

One of the more intriguing findings from the present work is that the strong anti-tumor activity for F10 occurs with no apparent damage to normal brain tissue, including brain tissue proximal to the tumor mass. These in vivo results are consistent with the lack of toxicity for F10 towards primary neuronal cultures (Fig. 2) and contrast sharply with the cytotoxicity of F10 towards GBM cells (Fig. 1). Our previous studies demonstrated that F10 selectively targets replicating cells with the lethal lesions being trapped Top1CC [11]. As mature neuronal cells have low proliferative capacity, the lack of toxicity for F10 towards these cells is not unexpected. In contrast, the conventional FP 5-FU is considerably more cytotoxic towards primary neuronal cultures than F10 (Fig. 2), likely as a consequence of RNA-mediated effects or metabolites [23] such as alpha-fluoro-beta-alanine (FBAL) (Fig. 1b) that are highly neurotoxic [24]. Nonetheless, establishing that the preferential cytotoxicity of F10 towards malignant cells in vitro can be achieved in vivo is a significant accomplishment that establishes the feasibility of using F10 for treatment of GBM in humans, particularly with local administration. To date, conventional FPs have displayed limited utility for GBM treatment in humans [25]. Thus, the promising activity towards GBM observed in the present studies with F10 indicates this novel polymeric FP may be fundamentally different from other FPs in this regard.

## Electronic supplementary material

Below is the link to the electronic supplementary material.

Supplementary material 1 (PDF 75 kb)
