# Supplementary material for: Selective anti-tumor activity of the novel fluoropyrimidine polymer F10 towards G48a orthotopic GBM tumors
Source: J Neurooncol. 2013 Dec 18;116(3):447–54. doi: 10.1007/s11060-013-1321-1 (PMC3905194; doi:10.1007/s11060-013-1321-1)
Supplement: Supplementary file 1 — Supplementary material 1 (PDF 75 kb) [file 11060_2013_1321_MOESM1_ESM.pdf]

Supplementary Material

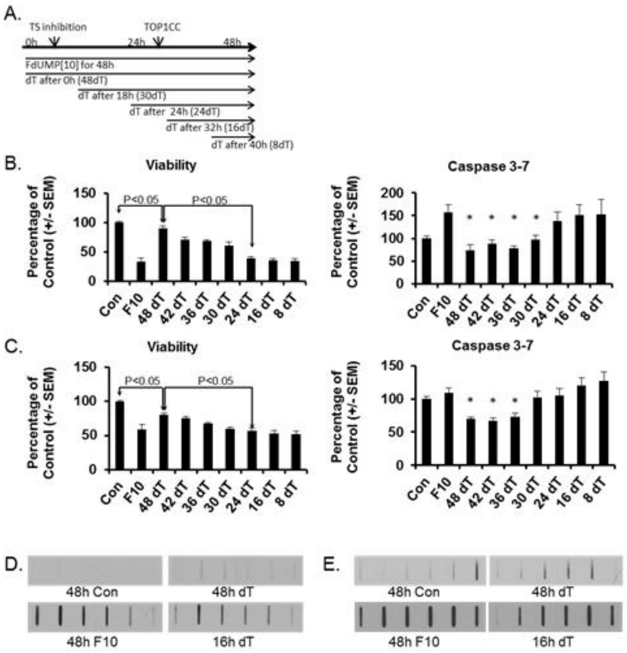

Supplementary Fig. 1. **F10 Cytotoxic and apoptotic effects are rescued by exogenous Thy only during first replicative cycle**. (a) Timing of Thy-rescue experiments; (b,c) Effect of F10 for 48 h treatment towards (b) SNB19 and (c) U251 cells. Exogenous Thy (dT - 80 μM) was added as a potential rescue agent for the indicated times from end of treatment. For each cell line, the graph on the left represents cell viability while the graph on the right represents apoptosis. 48 h dT rescue in U251 cells was not complete and 24 h rescue was significantly

reduced relative to 48 h rescue ($p < 0.05$). Apoptosis was significantly reduced by Thy rescue for these timepoints indicated with a * ($p < 0.05$). Thy rescue effects are limited to the first 24 h of F10 treatment. (d,e) In vivo complex of enzyme (ICE) bioassay results demonstrating that F10 induces Top1CC formation in (d) SNB-19 and (e) U-251 MG cells. Exogenous Thy rescues Top1CC if co-administered with F10 for 48 h but is not effective at preventing Top1CC formation if administered during the final 16 h of treatment after cells had committed to or undergone DNA replication and mitosis.

Supplementary Methods:

Tissue Processing and Immunohistochemistry. Endogenous peroxidase activity and non-specific biotin was quenched with Peroxide Blocking Kit and Biotin Blocking Kit, respectively (ScyTek Laboatories, Logan, UT). Antigen retrieval was performed with 10 mM sodium citrate buffer, pH 6.0, by microwaving for 5 min. Slides were blocked with SuperBlock (ScyTek) and incubated with polyclonal EphA2 antibody (R&D Systems, Minneapolis, MN) overnight at 4 °C. Slides were washed with PBS followed by incubation with biotinylated anti-goat antibody for 15 min, then Avidin-HRP for 20 min (ScyTek). Visualization with NovaRed (Vector Labs) was performed and allowed to develop for 2-10 min. Slides were counterstained with hematoxylin for 1 min, dehydrated, and mounted with Permount (Fisher). Additionally, sections were processed for hematoxylin-and-eosin staining using standard procedures.

Cortical Neuronal Cultures: In brief, cortices were isolated from embryonic day 18 mice. The tissue was further dissected into smaller pieces. After washes in cold PBS without Ca^{2+}/Mg^{2+}, tissue was incubated in 0.05% trypsin in PBS without Ca^{2+}/Mg^{2+}. Tissue was then dissociated in

NB media using a blue-tip pipette. Dissociated cells were layered onto a 4% BSA cushion to remove debris. Cells were plated in NB media at a density 4 x 10^4 cells/well in a 24-well dish. After 24 hours, media was removed and replaced with fresh media with or without 5-FU or F10. After 72 hours in culture, surviving neurons were counted. To be counted, a cell had to have a phase-bright cell body and at least one neurite that was at least two times the diameter of the soma
